# A kinematic and kinetic dataset of 18 above-knee amputees walking at various speeds

**DOI:** 10.1038/s41597-020-0494-7

**Published:** 2020-05-21

**Authors:** Sarah Hood, Marshall K. Ishmael, Andrew Gunnell, K. B. Foreman, Tommaso Lenzi

**Affiliations:** 1 Department of Mechanical Engineering and Utah Robotics Center, University of Utah, Salt Lake City, UT USA; 2 Department of Physical Therapy and Athletic Training, University of Utah, Salt Lake City, UT USA

**Keywords:** Biomedical engineering, Translational research

## Abstract

Motion capture is necessary to quantify gait deviations in individuals with lower-limb amputations. However, access to the patient population and the necessary equipment is limited. Here we present the first open biomechanics dataset for 18 individuals with unilateral above-knee amputations walking at different speeds. Based on their ability to comfortably walk at 0.8 m/s, subjects were divided into two groups, namely K2 and K3. The K2 group walked at [0.4, 0.5, 0.6, 0.7, 0.8] m/s; the K3 group walked at [0.6, 0.8, 1.0, 1.2, 1.4] m/s. Full-body biomechanics was collected using a 10-camera motion capture system and a fully instrumented treadmill. The presented open dataset will enable (i) clinicians to understand the biomechanical demand required to walk with a knee and ankle prosthesis at various speeds, (ii) researchers in biomechanics to gain new insights into the gait deviations of individuals with above-knee amputations, and (iii) engineers to improve prosthesis design and function.

## Abstract (summary)

**Table:** 

Measurement(s)	Biomechanics • Kinetics • kinematics • walking • Above-Knee Amputation • transfemoral amputation
Technology Type(s)	motion capture system • treadmill
Factor Type(s)	Medicare Functional Classification Level • walking speed
Sample Characteristic - Organism	Homo sapiens

Machine-accessible metadata file describing the reported data: 10.6084/m9.figshare.12221543

## Background & Summary

Motion capture has become an essential part of gait analysis. This experimental technique enables researchers to quantify how humans move and interact with the environment. Motion capture is fundamental for clinical gait analysis—the study of pathological gait^1,2^. The quantitative information gained from motion capture can assist in understanding the etiology of gait abnormalities, treatment decision making, and designing new therapeutic interventions such as walking aids and assistive devices^2–6^. Unfortunately, motion capture requires dedicated space, expensive equipment, specialized technicians, and significant amounts of time for both data collection and data processing. Furthermore, access to the patient population may be limited for most researchers interested in the interpretation of the biomechanics data. Thus, making gait datasets open is crucial to minimizing the cost and maximizing the impact of gait analysis.

Gait analysis is critical to assess the mobility level of individuals with gait impairment. The potential for community mobility in individuals with lower-limb amputations is internationally rated using a mobility grade scale^7–9^. The mobility grade assigned to an individual affects treatment decision making. For example, in the US, the Medicare Functional Classification Level, K-Level^10,11^, is used by Medicare, Medicaid, and many other private insurance companies to determine eligibility for payment or reimbursement of prosthetic technologies^12^. Currently, this classification is largely based on clinical outcome tests focusing on walking speed, as well as on the physician’s subjective assessment^13,14^. However, the relationship between K-level and pathological gait biomechanics is not clear^11,12,15^. This relationship is particularly difficult to address due to the high variability within this population. Motion capture has the potential to address this challenge by providing a quantitative understanding of the relationship between biomechanics and K-levels. Although many research studies have focused on the gait analysis of individuals with above-knee amputations, there is no open biomechanics dataset for this patient population.

In this paper, we present an open dataset of above-knee amputee biomechanics. The proposed dataset has been established in 18 individuals with unilateral above-knee amputations. Nine subjects were classified as full community ambulators (K3), and nine subjects were classified as limited community ambulators (K2). The classification was performed based on the subject’s ability to walk comfortably at or above 0.8 m/s^16^. The proposed dataset includes kinematics and kinetics collected while walking on an instrumented treadmill at five different speeds. A different set of speeds were used for the two groups based on K-level. The raw data recorded consisted of three-dimensional trajectories of 61 cutaneous reflective markers spread over the whole body, and the kinetics collected from the force plates, all synchronized and recorded in real time. For each subject, we collected five walking trials for each walking speed condition plus three calibration trials. This open biomechanics dataset will provide a new resource for clinicians to make treatment decisions^3,6^, for therapists to select appropriate therapeutic interventions^4,5^, and for researchers to design better prosthesis technologies^17–20^.

## Methods

We enrolled a total of 18 subjects with an above-knee amputation in this study. All subjects had received a unilateral above-knee amputation at least one year prior to the enrolment, have used a prescribed prosthesis for at least six months, and self-reported using the prosthesis for at least 3 hours a day. Table 1 shows the demographic data of the subjects. The experimental protocol for this study was approved by the University of Utah Institutional Review Board. All subjects provided written informed consent, including written permission to publish photos and videos of the experiment.

**Table 1:** Relevant Subject Information.

Subject Code	Age(yrs)	Gender	Mass(kg)	Height(m)	Amputation side	Etiology	Age of Amputation (yrs)	K-Level	Prescribed Prosthesis		Socket Suspension	Training? (#)	Hand-rails?
									Knee	Ankle			
TF01*	26	Male	64.9	1.78	Right	Traumatic	5	K3	Plie FI	AllPro FI	Suction	No	No
TF02**	79	Male	126.1	1.75	Right	Infection	1	K2	C-Leg Obk	Triton Obk	Lanyard	Yes (2)	Yes, All
TF05	72	Male	79.4	1.65	Left	Traumatic	4	K2	C-Leg Obk	Trition Low Profile Obk	Suction	No	No
TF06	60	Male	86.6	1.70	Left	Dysvascular	2	K2	C-Leg Obk	Kinterra FI	Lanyard	Yes (3)	Yes, All
TF07***	49	Male	102.1	1.91	Left	Traumatic	10	K3	C-Leg Obk	Triton Obk	Pin Lock	No	No
TF08	42	Male	95.3	1.85	Right	Traumatic	6	K3	Rheo Os	AllPro FI	Suction	No	No
TF09	65	Male	69.4	1.70	Left	Traumatic	2	K2	C-Leg Obk	Trias Obk	Suction	No	No
TF10	57	Female	58.5	1.65	Left	Traumatic	11	K2	C-Leg Obk	Trias Obk	Suction	No	Yes, All
TF11	51	Male	70.3	1.68	Right	Traumatic	33	K3	C-Leg Obk	Trias Obk	Suction	No	No
TF12	59	Male	99.8	1.83	Left	Traumatic	16	K2	C-Leg Obk	Trias Obk	Lanyard	Yes (1)	No
TF13	61	Male	88.5	1.88	Left	Traumatic	3	K3	Rheo Os	Proflex XC Os	Vacuum	No	Yes, LS
TF14	51	Male	108.9	1.73	Right	Traumatic	3	K2	Genium X3 Obk	Triton Obk	Lanyard	No	Yes, All
TF15	23	Female	68.0	1.75	Right	Traumatic	5	K3	Plie FI	Proflex XC Os	Suction	No	Yes, LS
TF16	36	Male	100.2	1.80	Left	Traumatic	8	K3	C-Leg Obk	AllPro FI	Suction	No	No
TF17	38	Male	104.3	1.91	Left	Traumatic	33	K3	Plie FI	Soleus ClgPk	Suction	No	No
TF18	69	Male	129.3	1.73	Right	Traumatic	50	K2	3R46 Obk	Renegade FI	Suction	No	Yes
TF19	30	Female	59.0	1.60	Left	Traumatic	10	K3	3R80 Obk	AllPro FI	Lanyard	No	No
TF20	59	Male	120.2	1.78	Left	Traumatic	42	K2	C-Leg Obk	Action Obk	Suction	No	Yes

Obk – Ottobock, FI – Freedom Innovations LLC, Os – Ossur, ClgPk – College Park Industries, LS – subject only used handrails on the last speed.

^*^Subject only has 4 walking trials for speeds 0.6, 0.8, 1.0 m/s

^**^Subject was unable to walk at the last speed of 0.8 m/s.

^***^Subject only has 4 walking trials for speed 0.6 m/s.

Upon enrolment, subjects were asked about their previous experience with walking on a treadmill. Subjects who reported using the treadmill regularly were asked to report their maximum comfortable treadmill speed and reliance on handrails during treadmill use. If a subject reported little to no experience walking on a treadmill with a prosthesis, they were provided training. Training consisted of the subject walking on the treadmill for 2–5 minute intervals with periods of rest in between each interval. During each training session, the experimenter started at the slowest speed of the treadmill (i.e., 0.2 m/s) and slowly incremented up until the subject reported that the speed was their maximum comfortable treadmill speed. Each training session lasted less than 2 hours. Weekly training sessions were conducted for three subjects, see Table 1, until no further improvements in comfortable walking speed were observed.

Subjects were divided into two groups based on the observed and reported maximum comfortable walking speed as well as their reliance on handrails. The walking speed of 0.8 m/s is commonly used as a threshold for determining if a subject is considered a limited community ambulator (K2) or full community ambulator (K3)^16^. Thus, for this study, we defined two subject groups (i.e., K2 and K3 group) based on the threshold speed of 0.8 m/s. Specifically, subjects were assigned to the K3 group if they could walk without using handrails at speeds up to 1.2 m/s. If a subject required the assistance of handrails for any speed above 0.8 m/s or their maximum walking speed was 0.8 m/s or lower, they were assigned to the K2 group. Different speed ranges were used during data collection for the two groups. The K2 group walked at [0.4, 0.5, 0.6, 0.7, 0.8] m/s; the K3 group walked at [0.6, 0.8, 1.0, 1.2, 1.4] m/s. An equal number of subjects were assigned to the K3 and K2 groups, Table 1.

Data collection was performed in a single session lasting no more than two hours. The experimental protocol comprised four sequential steps as described below.

1. *System Initialization*. Motion capture was performed using a 10-camera Vicon system (Vicon Motion Systems Ltd; Oxford, UK) and a split-belt (20in width) Bertec Fully Instrumented Treadmill (Bertec Co; Columbus, OH). The treadmill has a frontal handrail and two lateral handrails that are not instrumented. The motion capture cameras and the instrumented treadmill were initialized following the instructions provided by the manufacturer^21^. The initialization protocol included calibration of the cameras within the capture volume^21^, leveling the treadmill^21^, setting the volume origin^21^, and zeroing the force plates of the treadmill^21^.
2. *Preparation of the subject*. The subject wore tight fitted clothing and comfortable walking shoes. Reflective markers (14-mm diameter, 2-mm base) were placed on the subject following a modified Plug-in-Gait Model^22–26^ shown in Fig. 1 and listed in Table 1. The thigh and shank marker clusters were attached to the sound leg using a wrap and attached to the amputation side by directly placing the markers on the subject’s prosthesis and socket. An elastic wrap was placed over the abdomen to prevent the harness from blocking pelvic markers and to minimize soft tissue artifact. An upper-body harness was securely fastened to the subject. The subject was then directed onto the treadmill and the harness was connected to an overhead support system.

**Fig. 1:**
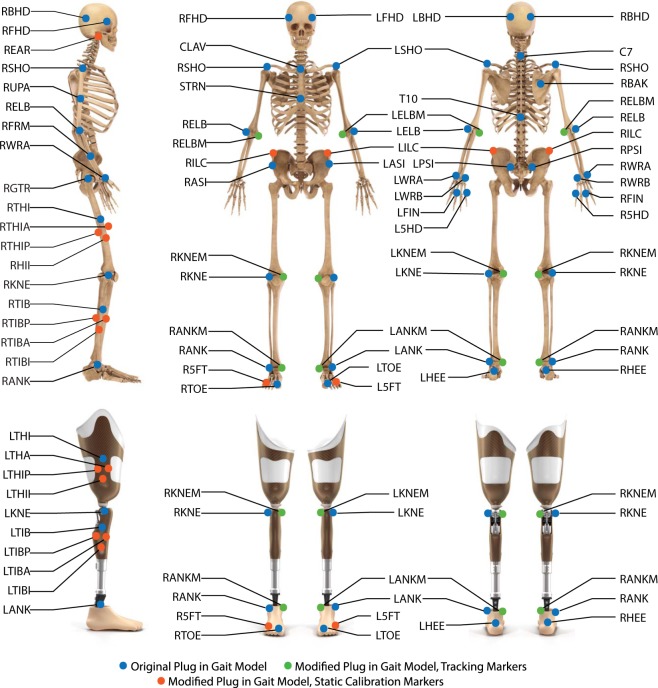
Locations and names of the markers in the modified Plug In Gait model. Markers used in both the original and modified Plug In Gait model are shown in blue. Markers used only in the modified Plug In Gait model are shown in orange for tracking and green for static calibration.
3. *Calibration Routines*. Three calibration routines were performed with the subject on the treadmill. *Static Calibration*: the subject was asked to stand still for five seconds with legs shoulder-width apart and arms out in front with elbows slightly bent^27^. Static calibration markers are removed after static calibration is completed. *Functional Calibration*: the subject was brought up to the middle speed within the classification and were recorded walking for approximately five strides^27^. *Joint Center Calibration*: the subject was asked to swing each leg, individually, in a clock pattern from noon to six or six to noon, depending on the side. Then, the subject performed two squats to bend the knee joint of the prosthesis^27^.
4. *Walking trials*. The subject walked at each speed in the assigned group starting from the slower speed. Subjects were instructed to hold on to the treadmill’s handrails during acceleration and deceleration of the treadmill. After the treadmill reached the desired speed, the experimenter encouraged the subject to walk without using the handrails. Subjects that were not able to walk without the support of handrails were noted by the experimenter (Table 1). For each treadmill speed, five trials of at least ten strides were recorded. If the subject crossed the force plates during data acquisition, the recording was stopped, and a new trial was recorded. After five trials of ten strides were successfully performed, the experimenter stopped the treadmill and the subject was given time to rest. The same protocol was repeated for the five different speeds specified in the subject group.

Marker trajectories and ground reaction force data were synchronized, recorded, and pre-processed using Vicon Nexus 2 software. Marker trajectory data was collected at 200 Hz and the ground reaction force data at 1000 Hz. Pre-processing consisted of labeling the markers, defining segments and calculating segment dimensions using the static and functional calibration routines. Once the markers were labeled, gaps in the marker trajectories were filled using rigid-body, spline, and pattern fill algorithms^21^. Gait events such as heel strike and toe off were detected and marked^21^. After the pre-processing was completed, the marker trajectories, force plate analog data and gait events were imported into Visual 3D software (C-Motion Inc, Germantown, Maryland). A low-pass Butterworth filter with a cut-off frequency of 6 Hz was applied for the marker trajectories. A low-pass Butterworth filter with cut-off frequency of 15 Hz was applied for the analog force plate data. The cut-off frequency for the low-pass Butterworth filter was determined using residual analysis^28^. Low-pass filtering was applied to the marker trajectories and analog force plate data before further analysis was performed.

To calculate the kinetics and kinematics, some anthropometric data including joint center of rotations were approximated for each subject within Visual 3D. The approximation of segment dimensions was determined from the modified Plug-in-Gait model using the static calibration file.

Dempster’s^29^ and Hanavan’s^30^ assumptions were used in combination with the subject’s reported weight (including the prosthesis) and segment dimension approximations to determine each subject’s mass, center of mass, and inertial properties of each segment. While these assumptions are commonly used in the field, other sources for anthropometric and inertial assumptions, such as De Leva^31^ or Zatziorsky^32^, can be applied to the provided marker trajectory data. Notably, a prosthesis cannot be accurately modeled using the assumptions made for an able-bodied individual^33^. Thus, the kinetics and kinematics were calculated using the assumptions that prosthesis side shank weight is 1/3 of the assumed able-bodied shank weight, and the center of mass is 25% below the top of the shank after previous studies^34,35^. Although the prostheses center of mass and inertial properties can be measured experimentally, this approach requires dedicated, custom equipment^36^ and was found not statistically different from assumptions we have used^34,35^. Starting from the specific prostheses components reported in Table 1, researchers may be able find experimental approximations of the center of mass and inertial properties and apply them to the provided marker trajectory data.

The knee joint center of rotation for both the prosthesis and sound side was calculated using the Symmetrical Axis of Rotation Analysis, SARA^37,38^, from the joint center calibration file. Noticeable error was observed when using the Symmetrical Center of Rotation Estimation, SCORE, for calculating the hip joint center of rotation. Upon analysis we concluded that this error correlated to movement at the socket-tissue interface. As a result, the Charnwood Dynamics Model, CODA^39,40^, was used to find the hip joint center of rotation. Using the joint center of rotations and the anthropometric assumptions, we computed the kinetics and kinematics of the ankle, knee and hip for both the contralateral (sound side) and ipsilateral (prosthesis side) side. Each subject’s use of the handrails, which were not instrumented, has been noted in Table 1. The kinetics and kinematics were calculated in Visual 3D and then exported into MATLAB (Mathworks, Natick, MA) data files for each subject.

## Data Records

**Fig. 2:**
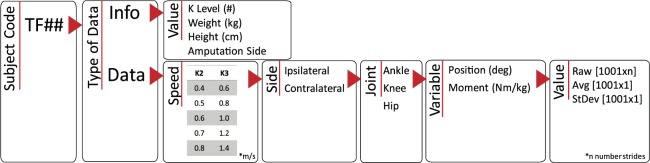
Matlab structure in which the kinetic and kinematic data is saved.

All data is made open using Figshare^41^. The subjects have been identified with an alphanumeric code using the format TF## noted for transfemoral amputation (TF) followed by their identification number (##). The de-identified subject information is stored in the Excel file named “*Subject Information”*. This file contains the alphanumeric codes, anthropometric data, ages, etiology of amputation, and prescribed prosthesis information for all subjects. The motion capture data has been grouped into folders. Each folder contains the data related to one subject and is named after the subject’s alphanumeric code. Within each subject folder there are two workspace folders, the Vicon Workspace and the Matlab Workspace. The Vicon Workspace contains the static, functional, and joint center calibration collections, as well as all the dynamic trials for the subject. The dynamic trials are noted as speed_## for the speed at which the subject walked for that collection, followed by the index of the collection at that speed. The speed was noted as 0p4 or 1p4, where *p* is used to replace the period in the naming of the speed using the units of m/s. For example, 0.4 m/s is coded as 0p4_##. The indexing of the collection for each speed is not sequential for all speeds, for example indexing of [01, 02, 03, 05, 06] where the index of [04] is dropped. This nonsequential indexing is due to the removal of collections with errors, such as a marker dropped, the subject crossed force plates during a step, or the subject requested an early rest. The Vicon workspace contains all the information about the collection and stores both the raw, unprocessed data from each collection, as well as the post-processed data after filling was completed. Vicon also creates a .c3d file for use in other software programs such as Visual 3D for further analysis and modeling. The marker trajectory data is provided so that any desired anthropometric and inertial assumptions can be applied to the data, including assumptions for the prosthesis. The MATLAB Workspace contains a data file for each subject with the processed kinetic and kinematic profiles. The MATLAB database is organized into a structure as shown in Fig. 2. The stride time is normalized to 1001 samples. All the strides for a given speed and side are saved as the raw data, along with the average and standard deviation across all strides. The folder *Matlab Plotting Functions* contains an *example_plotting_script* for how to use the plotting functions also contained in the folder. These plotting functions are built to compare speeds for a given variable (*plot_allspeeds_pos_comp_sides*), or to compare the ipsilateral and contralateral sides for a given subject (*plot_eachspeed_comp_sides*). The Vicon and Matlab workspaces provide a comprehensive dataset of the experiments by including raw marker data and processed joint biomechanics.

## Technical Validation

### Calibration of the motion capture volume

The capture volume consisted of 10 Vicon Cameras and a dual-belt fully instrumented Bertec Treadmill. Vicon Nexus software was used to synchronize the equipment and to perform the calibrations. As discussed in the Methods section, the capture volume was calibrated before each collection following the manufacturers recommended procedure^21^. This initialization protocol included calibration of the cameras within the capture volume^21^, leveling the treadmill^21^, setting the volume origin^21^, and zeroing the force plates of the treadmill^21^. For calibration of the cameras, a 5-marker wand and L-frame was used with 1000 refinement frames and 500 DV calibration frames. The global  error of each camera displays the calibration error in mm and is saved in the ‘*.xcp*’ file for each recorded trail.

### 3D trajectories of reflective markers

The 3D trajectories for each of the reflective markers were fully reconstructed to alleviate any gaps in the trajectories. This filling was performed on all walking trials and the joint center calibration file. Note that no filling was performed on the static and functional calibration files. Image error is reflective of the 3D reconstruction accuracy and is saved for each camera in the ‘*.xcp*’ file for each recorded trial. All reconstruction and filling have been saved and stored in the ‘*.history*’ file.

### Modified Plug-in-Gait model

A modified Plug-in-Gait Model was used for all experiments. This marker set used 67 reflective markers as listed in Table 2 to define 15 body segments (i.e., 2 feet, 2 shanks, 2 thighs, pelvis, trunk, head, 2 hands, 2 forearms, 2 arms). The exact location of the markers has been shown in Fig. 1. Compared to the standard Plug-in Gait model recommended by Vicon Nexus^22^, our modified Plug-in-Gait model used an additional 28 markers^23,24^. These additional markers were helpful in pre-processing to perform rigid-body-filling with markers specific to the segment without overlap across segments, minimizing inter-segment dependency^24^. Additional static calibration markers were also placed on the medial joints to measure joint width (e.g. markers on the medial and lateral sides of the knee used to calculated knee joint width). Notably, the medial markers were only placed for the static calibration file and then were removed for the rest of the protocol. The modified Plug-in-Gait Model was developed by adapting principles suggested in other models^25,26^.

**Table 2:** Detailed Marker Information: Description, Type, Placement.

Name	Description	Marker Type	Placement
LFHD	Left forehead	Tracking	Left temple
RFHD	Right Forehead	Tracking	Right temple
LBHD	Left Back Head	Tracking	Left posterior of the head
RBHD	Right Back Head	Tracking	Right posterior of the head
LEAR	Left Ear	Tracking	Left ear lobe
REAR	Right Ear	Tracking	Right ear lobe
C7	C7	Tracking	7^th^ cervical vertebra
T10	T10	Tracking	10^th^ thoracic vertebra
CLAV	Clavicle	Tracking	Jugular notch
STRN	Sternum	Tracking	Xiphoid process
RBAK	Right Back	Tracking	Anywhere over right scapula
LSHO	Left Shoulder	Tracking	Acromio-clavicular joint
LUPA*	Left Upper Arm	Tracking	Upper lateral 1/3 surface of left upper arm
LELB	Left Elbow	Tracking	Left Lateral Epicondyle
LELBM	Left Elbow Medial	Static Calibration	Left medial epicondyle
LFRM*	Left Forearm	Tracking	Lower Lateral 1/3 surface of left forearm
LWRA	Left Wrist A	Tracking	Lateral of the left wrist on thumb side
LWRB	Left Wrist B	Tracking	Medial of the left wrist on 5^th^ digit side
LFIN	Left Finger	Tracking	Proximal knuckle of 3^rd^ digit of left hand
L5HD	Left 5^th^ digit	Tracking	Proximal knuckle of 5^th^ digit (MCP joint) of left hand
RSHO	Right Shoulder	Tracking	Acromio-clavicular joint
RUPA*	Right Upper Arm	Tracking	Upper lateral 1/3 surface of right upper arm
RELB	Right Elbow	Tracking	Right Lateral Epicondyle
RELBM	Right Elbow Medial	Static Calibration	Right medial epicondyle
RFRM*	Right Forearm	Tracking	Lower Lateral 1/3 surface of right forearm
RWRA	Right Wrist A	Tracking	Lateral of the right wrist on thumb side
RWRB	Right Wrist B	Tracking	Medial of the right wrist on 5^th^ digit side
RFIN	Right Finger	Tracking	Proximal knuckle of 3^rd^ digit of right hand
R5HD	Right 5^th^ digit	Tracking	Proximal knuckle of 5^th^ digit (MCP joint) of right hand
LASI	Left ASIS	Tracking	Left anterior superior iliac spine
RASI	Right ASIS	Tracking	Right anterior superior iliac spine
LPSI	Left PSIS	Tracking	Left posterior superior iliac spine
RPSI	Right PSIS	Tracking	Right posterior superior iliac spine
LILC	Left Iliac Crest	Tracking	Superior surface of the left iliac crest
RILC	Right Iliac Crest	Tracking	Superior surface of the right iliac crest
LGTR	Left Greater Trochanter	Tracking	Left greater trochanter
LTHI	Left Thigh	Tracking	Lateral surface of left thigh
LTHIA	Left Thigh Anterior	Tracking	On rigid cluster, anterior to LTHI
LTHII	Left Thigh Inferior	Tracking	On rigid cluster, inferior to LTHI
LTHIP	Left Thigh Posterior	Tracking	On rigid cluster, posterior to LTHI
LKNE	Left Knee	Tracking	Flexion-extension axis of left knee
LKNEM	Left Knee Medial	Static Calibration	Medial left knee joint
LTIB	Left Tibia	Tracking	Lateral surface of the left shank
LTIBA	Left Tibia Anterior	Tracking	On rigid cluster, anterior to LTIB
LTIBI	Left Tibia Inferior	Tracking	On rigid cluster, inferior to LTIB
LTIBP	Left Tibia Posterior	Tracking	On rigid cluster, posterior to LTIB
LANK	Left Ankle	Tracking	Lateral malleolus of left foot
LANKM	Left Ankle Medial	Static Calibration	Medial lateral malleolus of the left foot
LHEE	Left Heel	Tracking	Calcaneus at same height as LTOE
LTOE	Left Toe	Tracking	Second metatarsal head
L5FT	Left 5^th^ digit toe	Tracking	Posterior calcaneus at level of LTOE
RGTR	Right Greater Trochanter	Tracking	Right greater trochanter
RTHI	Right Thigh	Tracking	Lateral surface of right thigh
RTHIA	Right Thigh Anterior	Tracking	On rigid cluster, anterior to RTHI
RTHII	Right Thigh Inferior	Tracking	On rigid cluster, inferior to RTHI
RTHIP	Right Thigh Posterior	Tracking	On rigid cluster, posterior to RTHI
RKNE	Right Knee	Tracking	Flexion-extension axis of right knee
RKNEM	Right Knee Medial	Static Calibration	Medial right knee joint
RTIB	Right Tibia	Tracking	Lateral surface of the right shank
RTIBA	Right Tibia Anterior	Tracking	On rigid cluster, anterior to RTIB
RTIBI	Right Tibia Inferior	Tracking	On rigid cluster, inferior to RTIB
RTIBP	Right Tibia Posterior	Tracking	On rigid cluster, posterior to RTIB
RANK	Right Ankle	Tracking	Lateral malleolus of right foot
RANKM	Right Ankle Medial	Static Calibration	Medial lateral malleolus of right foot
RHEE	Right Heel	Tracking	Calcaneus at same height as RTOE
RTOE	Right Toe	Tracking	Second metatarsal head
R5FT	Right 5^th^ digit Toe	Tracking	Posterior calcaneus at level of RTOE

^*^Markers are intentionally placed offset in height between the left and the right side.
